# Low Dose Chronic Angiotensin II Induces Selective Senescence of Kidney Endothelial Cells

**DOI:** 10.3389/fcell.2021.782841

**Published:** 2021-12-08

**Authors:** Irfan Khan, Marcel O. Schmidt, Bhaskar Kallakury, Sidharth Jain, Shaunt Mehdikhani, Moshe Levi, Margarida Mendonca, William Welch, Anna T. Riegel, Christopher S. Wilcox, Anton Wellstein

**Affiliations:** ^1^ Lombardi Comprehensive Cancer Center, Department of Oncology, Georgetown University, Washington, DC, United States; ^2^ Division of Pathology, Georgetown University, Washington, DC, United States; ^3^ Department of Biochemistry and Molecular and Cellular Biology, Georgetown University, Washington, DC, United States; ^4^ Division of Nephrology and Hypertension, Kidney, and Vascular Research Center, Georgetown University, Washington, DC, United States

**Keywords:** angiotensin II, senescence, endothelial cells, INK-ATTAC, von Willebrand factor

## Abstract

Angiotensin II can cause oxidative stress and increased blood pressure that result in long term cardiovascular pathologies. Here we evaluated the contribution of cellular senescence to the effect of chronic exposure to low dose angiotensin II in a model that mimics long term tissue damage. We utilized the INK-ATTAC (p16^Ink4a^–Apoptosis Through Targeted Activation of Caspase 8) transgenic mouse model that allows for conditional elimination of p16^Ink4a^ -dependent senescent cells by administration of AP20187. Angiotensin II treatment for 3 weeks induced ATTAC transgene expression in kidneys but not in lung, spleen and brain tissues. In the kidneys increased expression of ATM, p15 and p21 matched with angiotensin II induction of senescence-associated secretory phenotype genes MMP3, FGF2, IGFBP2, and tPA. Senescent cells in the kidneys were identified as endothelial cells by detection of GFP expressed from the ATTAC transgene and increased expression of angiopoietin 2 and von Willebrand Factor, indicative of endothelial cell damage. Furthermore, angiotensin II induced expression of the inflammation-related glycoprotein versican and immune cell recruitment to the kidneys. AP20187-mediated elimination of p16-dependent senescent cells prevented physiologic, cellular and molecular responses to angiotensin II and provides mechanistic evidence of cellular senescence as a driver of angiotensin II effects.

## Introduction

The Renin-Angiotensin-Aldosterone system is implicated in hypertension, cardiovascular and kidney diseases, where angiotensin II causes vasoconstriction (Harrison-Bernard, 2009) and induces reactive oxygen species (ROS) leading to vascular inflammation and injury (Nataraj et al., 1999a; Guzik et al., 2007a). One cellular effect of ROS signaling is the induction of senescence that culminates in cell cycle arrest (Childs et al., 2015) and is associated with the upregulation of cell cycle proteins (p16^Ink4a^, p15^Ink4b^, p21^CIP1^). Other indicators of cellular senescence are DNA damage markers (gamma-H2Ax), chromatin alterations (decrease in lamin B1) and the induction of the SASP (senescence associated secretory phenotype) gene set (Demaria et al., 2014). SASP genes include proteases, cytokines, growth factors and chemokines (e.g. MCP-1, MIP-1a, IL-1a, IL-6, IGFPBs, FGFs, IL-8, and MMPs) that are known to promote the recruitment of inflammatory and immune cells to eliminate senescent cells (Childs et al., 2015; Sieben et al., 2018). The tissue response to the induction of cellular senescence, however, varies widely between cell types and tissues and also depends on the original insult (Childs et al., 2015). Most importantly, the p16 senescence induction is closely aligned with Rb and has been classified as the p16^ink4a^/Rb pathway (Kumari and Jat, 2021). When Rb is hyperphosphorylated by cyclin-dependent kinases 4/6 (CDK4/6), it can dissociate from E2F which allows for transcription of S phase genes and lead to progression of the cell cycle (Zhang et al., 2000). However, if p16 is expressed it can inhibit CDK4/6 which leads to Rb not becoming phosphorylated and thus staying bound to E2F causing transcriptional repression of cell cycle genes and leading to cellular senescence (Fischer and Muller, 2017).

**FIGURE 1:**
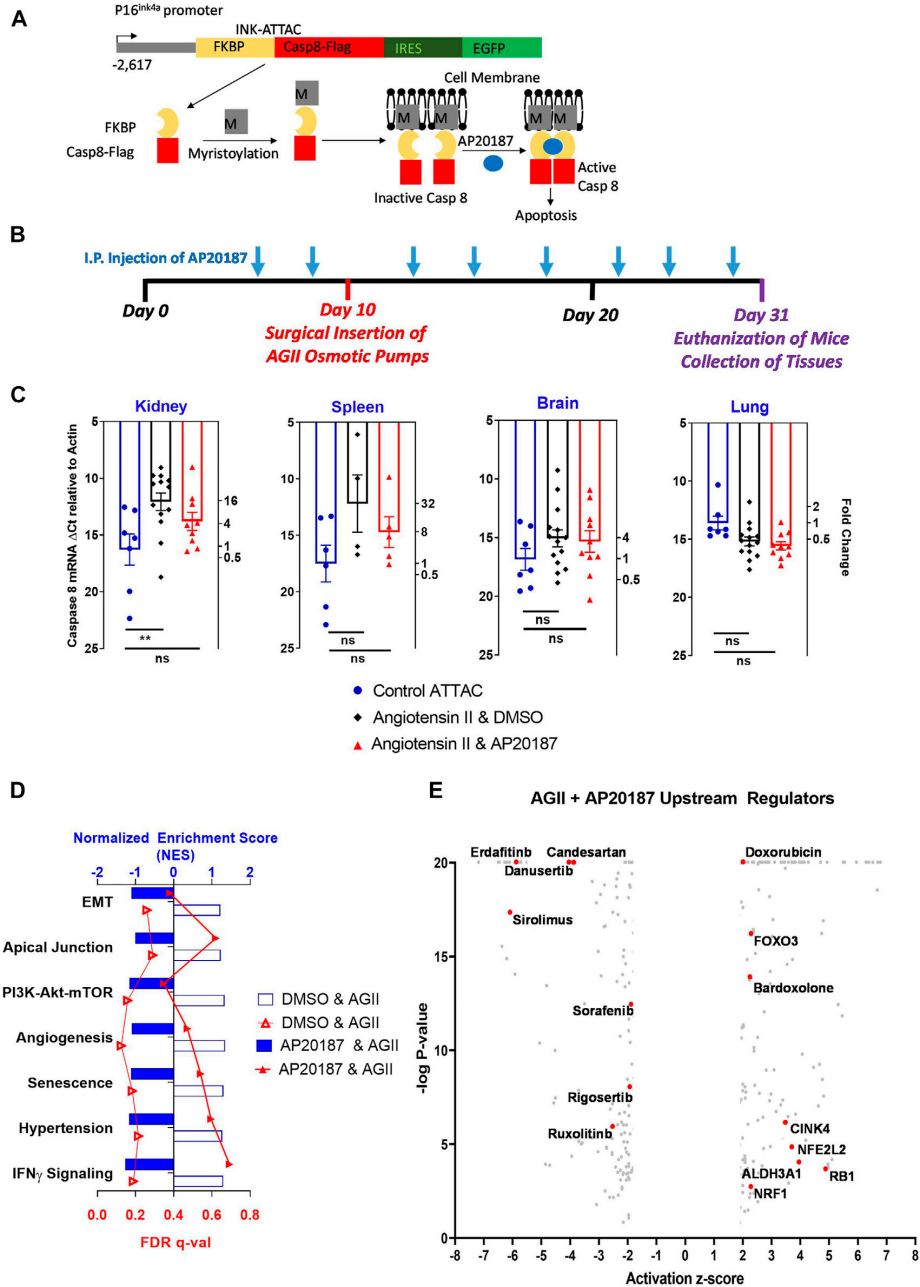
Low dose, chronic angiotensin II treatment effect in the INK-ATTAC mouse model. **(A)**, p16(Ink4a) promoter drives the transgenic ATTAC cassette. Model adapted from Baker et al. Nature 2011. Details in text. **(B)**, experimental design. Mice received vehicle or AP20187 (2 ug/g i. p.,) as indicated and angiotensin II (angiotensin II; 400 ng/kg/min *via* minipump). **(C)**, mRNA expression of transgenic human caspase 8 in different organs. Left ordinate: Ct-values from qRT-PCR relative to actin. Right ordinate: Fold change relative to the control group (note the log scale). **, *p* < 0.01; ns, not significant. **(D)**, major pathways impacted by the angiotensin II treatment in kidneys. RNA-seq data from vehicle or AP20187 treated mice were compared to mice at baseline (*n* = 3 in each group). Hallmark Pathways: EMT (Epithelial Mesenchymal Transition), Apical Junction, PI3k-Akt-mTor (PI3K-Akt-MTOR pathway), Angiogenesis. Gene Ontology Pathways: Senescence (Cellular Senescence), Hypertension (Renal System Process involved in regulation of systemic arterial blood pressure), IFNy signaling (Interferon Gamma mediated signaling pathway). **(E)**, upstream regulators identified by Ingenuity Pathway analysis. Angiotensin II treatment was compared to angiotensin II plus AP20187. Note: Supplementary Table S1 contains a complete listing and the numerical values.

To evaluate the contribution of senescence to the pathophysiology underlying different diseases two distinct transgenic mouse models have been used that take advantage of the central role of p16 as a major driver of senescence (reviewed in: 4, 57): The p16-3MR senescent mouse model from the Campisi laboratory (Demaria et al., 2014) and the INK-ATTAC mouse (p16^Ink4a^-Apoptosis through Targeted Activation of Caspase 8) from the van Deursen laboratory (Baker et al., 2011). Both rely on p16-dependent cellular senescence to induce a conditional, synthetically lethal transgene to eliminate senescent cell populations. The INK-ATTAC mouse used here contains the ATTAC transgene cassette downstream of a p16^Ink4a^ promoter. Activation of the p16^Ink4a^ promoter in senescent cells induces expression of a monomeric, inactive caspase 8 protein that can be dimerized and activated by exogenous administration of AP20187 inducing apoptosis and elimination of senescent cells (see Figure 1A). The caspase 8 produced by transcription of the ATTAC transgene is human and thus its expression levels will not be confused with endogenous mouse caspase 8. In addition, cells expressing the ATTAC transgene will also express green fluorescent protein (GFP) as it is present downstream of an IRES on the cassette and can be used to identify p16 positive cells by staining. The dimerizing ligand AP20187 has no known physiologic target and does not affect cells that lack expression of the ATTAC transgene (Baker et al., 2011). The unique benefit of the INK-ATTAC mouse model is that human caspase 8 mRNA levels can be used to identify which tissues are undergoing p16 induced senescence and staining for the GFP protein can then be used to detect senescent cells in those tissues. We applied both approaches here to screen potential target tissues for increased senescence and identify cell types in those tissues, with the AP20187 treatment group providing a negative control.

We subjected INK-ATTAC mice to a slow pressor infusion of angiotensin II that is known to cause a small increase in blood pressure, oxidative stress and cardiovascular damage (Kawada et al., 2002). Mice were treated with either AP20187 or vehicle during infusion of angiotensin II over 3 weeks and tissues before and after the different treatments were analyzed and compared. We observed p16^Ink4a^ induction in renal endothelial cells indicated by increased angiopoietin 2 expression, increased von Willebrand factor staining and expression and staining for GFP (part of the ATTAC transgene cassette). Angiotensin II also induced expression of senescence associated secretory phenotype (SASP) and inflammation-related genes in the kidneys as well as immune cell infiltration next to glomerular and peritubular capillaries. Most importantly, the elimination of senescent cells by AP20187 treatment prevented these effects of angiotensin II and provides mechanistic evidence of a rate-limiting contribution of cellular senescence.

## Methods

### Mice

INK-ATTAC C57/Bl6 mice were kindly provided by the van Deursen laboratory (Baker et al., 2011). Male mice aged 4–6 months were accommodated to the tail cuff plethysmography blood pressure procedure over 3 days. Baseline blood pressure readings were gathered at the same times over 3 days. Thereafter, mice were then divided into either vehicle (DMSO) or AP20187 (AP) (2 ug/g bodyweight) treatment groups (blue arrows Figure 1B). Blood pressure readings were taken immediately prior to drug delivery. Osmotic pumps containing angiotensin II were infused at 400 ng/kg/min for 21 days, while mice received either vehicle (DMSO) or AP. After 21 days, mice were euthanized for harvesting of brain, lung, kidney, and spleen. Segments of each tissue were collected in either RNAlater for qRT-PCR and RNA-seq, OCT for frozen sectioning or 10% formalin for paraffin sectioning.

### RNA Extraction and Real Time Quantitative PCR

Tissues in RNAlater were stored in −80°C. RNA extraction used the Qiagen RNeasy mini-kit. The concentration of RNA was estimated by Nanodrop and subsequently the sample was subjected to reverse transcriptase using the Bio-Rad I-script cDNA synthesis kit. Quantitative PCR was carried out using the Bio-rad iQ Sybr Green Supermix and the delta CT values for caspase 8 were subtracted from the baseline actin values. Graphs present caspase eight values with higher delta Ct values meaning lower expression of and lower delta Ct values signifying higher expression. On the right *Y*-axes the fold change of gene expression relative to control is shown.

### Immunohistochemistry of Formalin-Fixed Paraffin-Embedded Tissues

Tissues were embedded and sectioned by Georgetown Histology and Tissue Shared Resource Core. Slides were washed in Xylene and ethanol and boiled in 1X citrate buffer for 10 min for antigen retrieval and incubated in 3% H2O2 to eliminate endogenous peroxidase activity. Staining for CD45R was done using the Vector Laboratories Rat IgG Vectastain Elite ABC peroxidase Kit, while for, CD31, vWF, and CD68, the respective Rabbit IgG kit was used. For GFP, used the Vector Laboratories M.O.M (Mouse on Mouse) Immunodetection kit. Slides were blocked in 1x PBS for 30 min prior to incubation with the primary antibody at 4 C overnight. CD45R (ebioscience RA3-6B2) was done at 1/800 and 1/1,200 in the kidneys, vWF (Millipore AP7356) at 1/250 and 1/500, CD31 (Cell Signaling 77,699) at 1/250 and CD68 (Invitrogen PA5-78966) at 1/500 and 1/1,000. GFP (Invitrogen 332,600) was done at 1/5,000 and 1/10,000. CD3 staining was done by the GU HTSR with antigen retrieval done in Tris/EDTA pH 9 buffer and primary antibody (Agilent, A0452) incubation done at 1/200 for 1 h at room temperature. All antibodies were incubated with appropriate secondary antibodies for 30 min to 1 h and stains developed by DAB. Slides were subsequently stained in Hematoxylin and dehydrated in ethanol, washed in xylene and mounted on slides using cytoseal.

### Immunohistochemistry of Frozen Tissues

Frozen kidney tissues in OCT were stored in −80°C and were sectioned by American Histolabs and Georgetown Histology and Tissue Shared Resource Core. Slides underwent Beta-Galactosidase staining using the Cell Signaling Senesecence Beta-Galactosidase Staining Kit (9860S). Kidney samples were incubated in Beta-Gal staining solution at 37C in a humidified chamber for 48 h.

### RNA-Sequencing, Gene Set Enrichment Analysis, and Ingenuity Pathway Analysis

We used three independent kidney samples from each of the treatment groups for transcriptome-wide RNA expression analyses by RNA sequencing (RNA-seq). Sequencing was carried out at a depth of 40 million paired reads per sample with 150 bp sequencing length and run by Novogene Sacramento, CA. We processed files for differential expression analysis ussing the EdgeR package in R and then further analyzed for activated or inhibited pathways using GSEA and IPA (Subramanian et al., 2005). DMSO and angiotensin II samples were compared with control untreated samples and AP20187 and Angiotensin II samples for Hallmark, C2 and C5 (GO) pathways for differences in enrichment. The first comparison was used to select pathways highly enriched in the DMSO and angiotensin II samples and the second to test how those pathways were impacted by AP20187 and angiotensin II. Cutoff for significantly enriched pathways were FDR q-value < 0.25. The data were deposited to the GEO repository under GSE179195, will be publically accessible after publication of the manuscript and accessible upon request.

In previous studies we have used qRT-PCR for the measurement of mRNA abundance of individual genes and wished to compare the precision of our established qRT-PCR approach with the precision of RNA-seq. For this, we analyzed mRNA expression of five of the genes that are discussed in the Results section, i.e., ANGPT2, Cdkn1a, FGF2, IGFBP2 and MMP3. Quantitative PCR was carried out in duplicate using the Bio-rad iQ Sybr Green Supermix. Gene specific primer sequences for amplification of the respective cDNAs were obtained from the publically available MGH-Harvard Primer Bank (https://pga.mgh.harvard.edu/primerbank/). The Standard of Error of the Mean (SEM) of measurements of 3–4 independent biological samples of a given treatment group was then used as an indicator of the precision of the measurements. Across the five genes for the three experimental groups the median SEM was 0.37 and the mean SEM was 0.40. For the RNAseq of the same genes, we observed a mean SEM of 0.072 and a median SEM of 0.075. Thus, the RNAseq data showed a ∼ 5-fold smaller variation than qRT-PCR quantitation allowing for the detection of smaller differences in expression. The digital nature of RNA-seq produces absolute expression values based on the number of sequence reads and thus enables detection of subtle changes in expression. Direct sequence read counts also avoid the pitfalls of amplification steps required for qRT-PCR.

### Statistics

**FIGURE 2:**
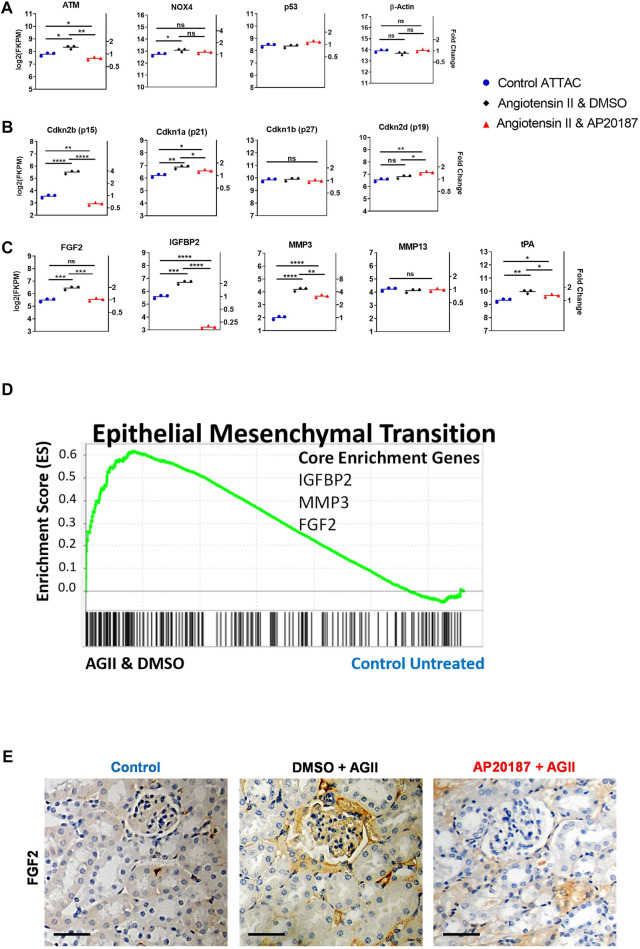
Impact of low dose, chronic angiotensin II treatment on senescence related pathways—RNA-seq analysis of kidneys. A to C, comparative expression of different signature mRNAs. Absolute expression (FKPM; **left axes**) and fold change relative to the control group **(** **right axes** **)** are shown (note the log scale of the ordinates). **(A)**, oxidative stress (NOX4), DNA damage (ATM, p53) and a control gene (beta-actin); **(B)**, Cyclin-dependent kinase inhibitor genes that control senescence pathways (p15, p21, p27, p19) Note: endogenous p16 mRNA was below detection by RNA-seq; **(C)**, SASP-related genes. *, *p* < 0.05; **, *p* < 0.01; ***, *p* < 0.001; **(D)**, GSEA enrichment for the EMT hallmark pathway with signature genes indicated; E, staining for FGF2 protein in the kidney cortex. Scale Bar is 0.1 mm. Three groups are compared i.e., control, angiotensin II + vehicle, angiotensin II + AP20187.

**FIGURE 3:**
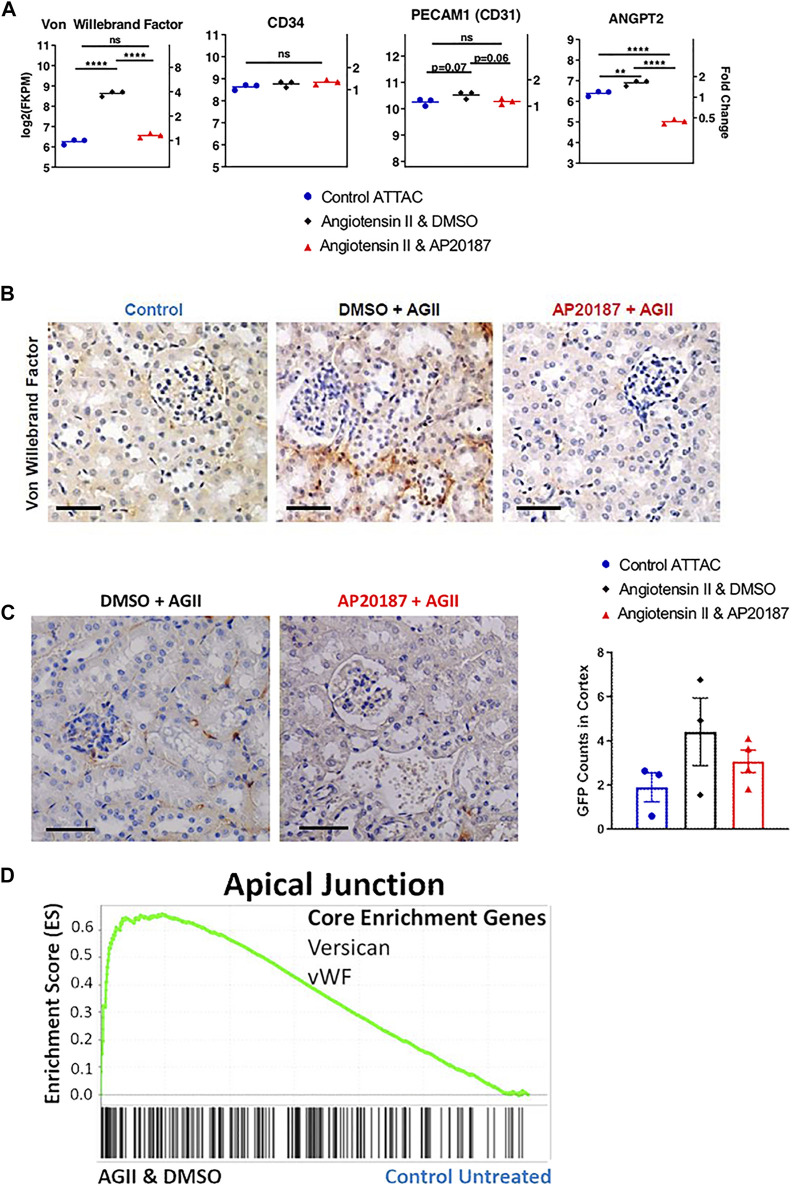
Impact of low dose, chronic angiotensin II treatment on endothelial-related genes and protein expression. **(A)**, endothelial cell markers (vWF, CD31, CD34) and ANGPT2 gene expression. **(B)**, vWF staining. **(C)**, staining for GFP expression from the ATTAC cassette (see Figure 1A). **(D)**, GSEA enrichment for the Apical Junction hallmark pathway with signature genes indicated; In A and B three groups are compared i.e., control, angiotensin II + vehicle, angiotensin II + AP20187. *, *p* < 0.05; **, *p* < 0.01; ***, *p* < 0.001; Scale Bars are 0.1 mm. Quantification was based on an average of 20 images of the kidney cortex per slide.

**FIGURE 4:**
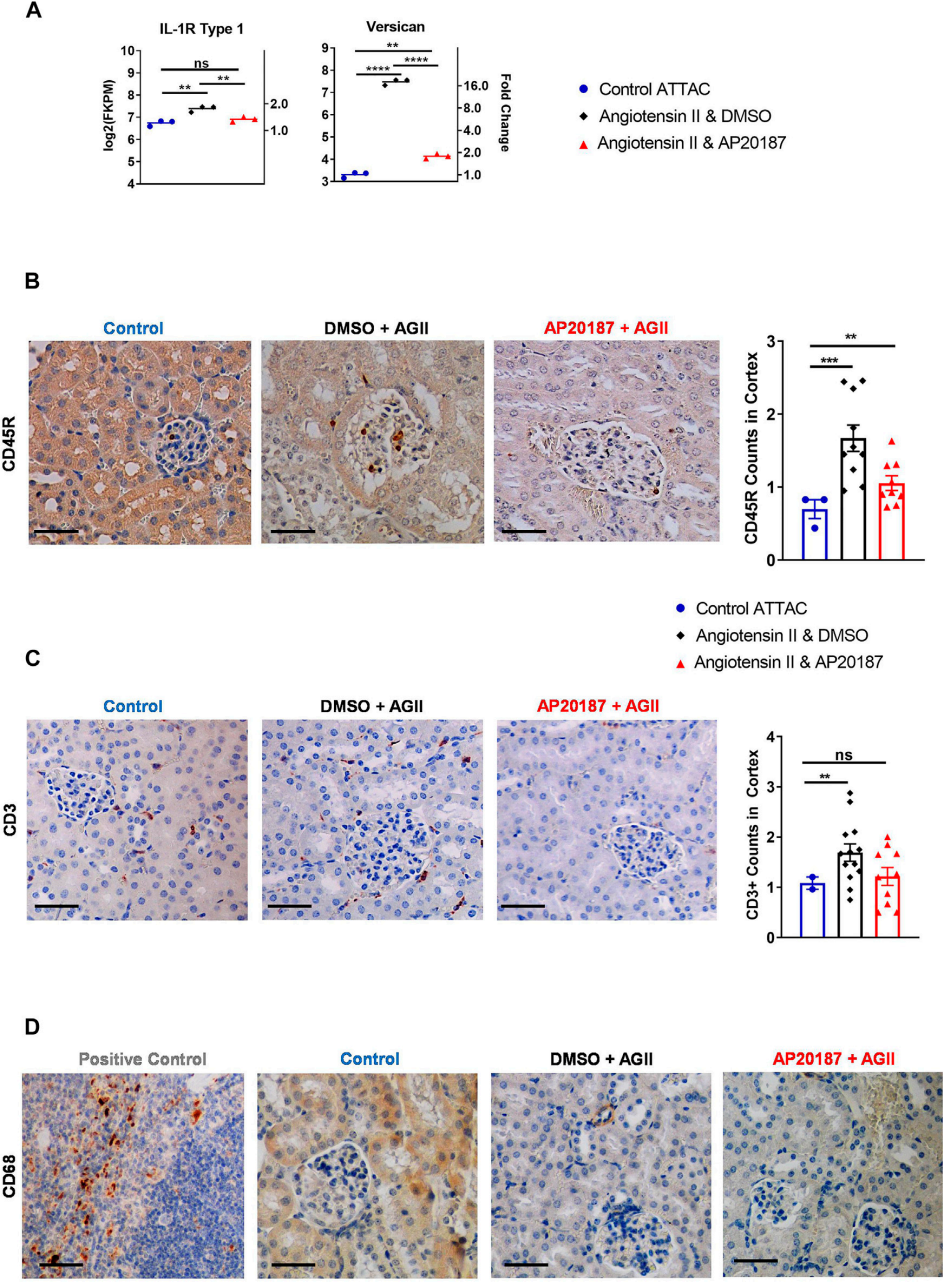
Inflammatory signaling and cell recruitment. **(A)**, IL-1R and versican mRNA expression. B to D, staining for inflammatory cell markers; CD45R staining and quantitation **(B)**; CD3 staining and quantitation **(C)**; CD68 staining **(D)**. Three groups are compared i.e., control, angiotensin II + vehicle, angiotensin II + AP20187. *, *p* < 0.05; **, *p* < 0.01; ***, *p* < 0.001. Quantification was based on an average of 20 images of the kidney cortex per slide.

Statistical analysis was done on GraphPad Prism 8. Statistical significance for Figure 1C, Figures 4B,C, S1A, S1B were analyzed by Mann Whitney one sample *t* test. Statistical significance for Figures 2A–C, Figure 3A, Figure 4A were analyzed by unpaired *t* test.

### Study Approval

Animal studies were approved by the Georgetown University IACUC (#2016-1113).

## Results

### Increased p16 Induction in Kidneys Upon Angiotensin II Treatment

Low dose prolonged infusion of angiotensin II is a classic stressor model to understand the initiation of borderline hypertension with the resulting long-term tissue damages (Kawada et al., 2002). Here we used the INK-ATTAC mouse model (Baker et al., 2011) (see Figure 1A) to assess whether increased senescence induction is observable over 3 weeks of angiotensin II and to what extent p16^Ink4a^ induced senescence is the rate-limiting mechanism. The expression of genes in the ATTAC transgene cassette is very low and undetectable in young adult mice and is not significantly modified by AP20187 treatment (Baker et al., 2011). We took advantage of this low background to screen different tissues for potential activation of the p16^Ink4a^ transgene during the low dose angiotensin II infusion. The activation of the ATTAC transgene was assessed from expression of the human caspase 8 coding sequence which does not share any homology with the endogenous mouse caspase 8 gene. The degree of reduction in expression of the human caspase 8 from the ATTAC cassette after AP20187 treatment is then used to assess the cellular apoptosis induced by the conditional activation of the caspase 8 fusion protein (Figure 1A). The expression of transgenic caspase 8 in the lungs, brains and spleens of mice infused for 3 weeks with angiotensin II was unchanged, whereas there was a significant 4-fold increase in expression in the kidneys that was restored to baseline by the AP20187 treatment (Figure 1C). Further analyses were thus confined to the kidneys.

### RNA Expression and Pathway Analysis in Kidneys

Transcriptome-wide analysis of kidneys from three different animals at baseline, three animals treated with angiotensin II, and three treated with angiotensin II plus AP20187 was carried out using unbiased RNA sequencing (RNA-seq) at a depth of 40 Mio paired sequence reads per sample and a sequencing length of 150 bp. This approach uses the expression of all detectable genes in a given sample (>15,000) to provide the amount of RNA in the sample for comparison of gene expression across samples. The RNA-seq data from this experimental series are available for download from the NIH repository (GEO at NCBI: GSE179195).

We initially analyzed the data set at a global level using the gene set enrichment analysis platform (GSEA; (Subramanian et al., 2005) (Figure 1D). Significantly altered pathways (FDR q-value < 0.25) in the kidneys of the angiotensin II treated group versus the baseline include cellular senescence and regulation of arterial blood pressure, while there was no significant change relative to baseline in the angiotensin II plus AP20187 treated group. In addition, the GSEA analysis showed genes controlling angiogenesis, epithelial-mesenchymal transition (EMT), PI3K-mTOR-Akt signaling, and apical junction as well as IFN-gamma pathways significantly enriched in the angiotensin II group but not the angiotensin II plus AP20187 group (Figure 1D and Supplementary Table S2). Furthermore, through Ingenuity Pathway Analysis upstream pathway regulators and their impact were identified (Figure 1E and Supplementary Table S1). Candesartan (angiotensin receptor blocker) regulated gene sets showed a highly significant (*p* = 2.2x10^−23^), negative 4.4-fold activation score (Supplementary Table S1) corroborating the specificity of gene expression changes due to angiotensin II exposure. Although the receptors for angiotensin II were below detection by RNA-seq, the above IPA finding for Candesartan highlights downstream regulatory pathways impacted by angiotensin and/or AP20187. Also, several FGF receptor kinase inhibitors (e.g., erdafitinib, danusertinib) regulated gene sets showed highly significant negative activation scores of >7 matching with the crosstalk between angiotensin II and FGFR signaling in the control of blood pressure (Tassi et al., 2018) and with the upregulation of the FGF2 ligand (see below). Sorafenib (PDGRF/VEGFR inhibitor) regulated gene sets showed negative activation which aligns with the angiotensin II, FGFR signaling and angiogenesis crosstalk. Rigosertib (PI3K pathway inhibitor) regulated genes were negatively regulated and this aligns with the GSEA analysis of the PI3K-mTOR-Akt signaling pathway (see above). Furthermore, gene sets regulated by sirolimus (mTOR inhibitor) and ruxolitinib (JAK2 inhibitior) exhibited negative activation scores matching with their known inhibition of SASP expression. On the other hand, gene sets controlled by inducers of senescence (e.g., doxorubicin, CINK4, FOXO3, RB1) showed positive activation scores which aligns with the increased senescence and SASP expression in mice exposed to angiotensin II (see below). Lastly, gene sets signifying activity of antioxidants (Bardoxolone, NRF1, NFE2L2) and ROS metabolizers (ALDH3A1) showed positive activation scores and thus corroborate the increased ROS production known to occur with angiotensin II.

### Senescence Pathways Impacted

A slow pressor infusion of angiotensin II induces oxidative stress (Kawada et al., 2002) that can account for the increased expression of ATM that is implicated in the DNA damage response pathway (Figure 2A). Its reversal by AP20187 treatment suggests that cells expressing the INK-ATTAC cassette and eliminated by the AP20187 treatment are the major responders to the oxidative damage and associated DNA damage. DNA damage is known to induce cellular senescence via cyclin dependent kinase inhibitor genes (Fischer and Muller, 2017; Stagni et al., 2021). Indeed Cdkn2B (p15^Ink4b^; Ref. (Hannon and Beach, 1994)) was significantly upregulated by 4-fold in the kidneys from angiotensin II treated animals (Figure 2B) but was completely reversed by AP20187. Cdkn1a (p21^Cip1^; Ref. 58) showed a similar pattern of changes although to a smaller extent while other senescence regulators i. e., p19 (Hashimoto et al., 2016) and p27 (Rukhland et al., 2016) were not impacted (Figure 2B).

The expression of genes for Senescence-Associated Secretory Phenotype (SASP) factors that were upregulated in the angiotensin II group and were reduced after addition of AP20187 treatment include tissue-type plasminogen activator (tPA), matrix metalloproteinase-3 (MMP-3), insulin growth factor binding protein-2 (IGFBP-2), and fibroblast growth factor-2 (FGF2) but other known SASP genes (Coppe et al., 2010; Kumari and Jat, 2021), e. g., MMP-13 were not affected (Figure 2C). Epithelial-mesenchymal transition (EMT) and fibrosis is a known response of the kidneys to chronic damage (Sheng and Zhuang, 2020). Gene sets that precede and control EMT (Smit and Peeper, 2010; Laberge et al., 2012) were also found significantly induced after angiotensin II treatment and reversed by the addition of AP20187 (Figure 1D, Figure 2D). The significant upregulation of FGF2 mRNA in the angiotensin II group relative to control and AP20187 (Figure 2C) resulted in detectable FGF2 protein staining of kidney tissues from the angiotensin II group that was barely detectable after AP20187 and in the controls. Staining was prominent in endothelial cells (Figure 2E) similar to the vWF and GFP staining shown below (see Figures 3B,C). Lastly, beta-galactosidase staining showed increased beta gal positive cells in the angiotensin II group that was decreased by addition of AP20187 (Supplementary Figure S2A).

### Endothelial Cells as Targets of Senescence Induction

An intriguing finding from the mRNA analysis in the kidney was the significant 4-fold increase of Von Willebrand factor (vWF) mRNA in the angiotensin II group and its reversal to control levels by adding AP20187 treatment (Figure 3A). This regulation was corroborated by staining for the vWF protein in the endothelial cells in the peritubular capillary network (Figure 3B). It is noteworthy that CD31 (PECAM1) and CD34 that are expressed preferentially in endothelial cells did not show differences in expression between the treatment groups (Figure 3A). This would further suggest that the upregulation in vWF is due to increased expression and secretion by damaged endothelial cells (Müller et al., 2002; Pusztaszeri et al., 2006). Also, the increased expression of angiopoietin 2 (ANGPT2), a growth factor secreted by damaged endothelial cells during inflammation (Figure 3A) (Kim et al., 2016; Kiss and Saharinen, 2017), supports this notion. Expression of ANGPT2 was reversed by AP20187 indicating that it is secreted specifically by senescent endothelial cells as ANGPT2 is part of the Weibel-Palade bodies which also contain vWF (Kim et al., 2016).

Finally, we took advantage of the GFP expression cassette that is part of the ATTAC transgene (see Figure 1A) to identify the cell type that expresses the transgene in the kidneys. Endothelial cells of the glomerular and peritubular capillary network showed a distinct staining for GFP in the angiotensin II group (Figure 3C) supporting the notion that endothelial cells respond to low dose angiotensin II with the induction of senescence pathways. Furthermore, we compared 5 µm thick serial sections of kidney tissues for CD31, vWF, and GFP staining to assess co-localization. From that comparison we found that there is overlap of vWF and GFP positive staining in the endothelial cells (green arrows in left panels of Supplementary Figure S2B). This coincident staining supports the notion that the increased vWF expression and staining are derived from endothelial cells undergoing senescence.

### Increased Immune Cell Recruitment in Angiotensin II Treated Kidneys

We next investigated whether the induction of senescence would impact the abundance of inflammatory and immune cell signatures or cells themselves (Langhi Prata et al., 2018; Kale et al., 2020). RNA-seq analysis demonstrated that angiotensin II increased expression of interleukin 1 Receptor 1 (IL-1R1) that was decreased by addition of AP20187 (Figure 4A). This receptor binds IL-1 leading to expression of SASP (Orjalo et al., 2009; Lau et al., 2019). Interestingly, versican, an inflammatory extracellular matrix proteoglycan showed a ∼16-fold upregulation after angiotensin II that decreased with the addition of AP20187 (Figure 4A) (Wight et al., 2020). Furthermore, the Apical Junction pathway of which versican is a signature gene was found significantly induced after angiotensin II treatment and reversed by the addition of AP20187 (Figure 3D).

At the cellular level angiotensin II increased CD45R + immune cells in the kidney glomerular and peritubular capillary network that was decreased by the addition of AP20187 (Figure 4B). An increase in T cells was indicated by CD3 staining (Figure 4C). Interestingly, CD68 staining did not show recruitment of macrophages in the kidneys. The spleen was used as a positive control for staining (Figure 4D).

## Discussion

Cellular senescence was recognized as a state of cell cycle arrest that prevents malignant transformation of damaged cells without causing cell death. Subsequently the understanding of the role of cellular senescence was expanded to the pathophysiology of Alzheimer’s Disease, Type 1 Diabetes, Pulmonary Fibrosis, Osteoarthritis, Cardiovascular and Kidney Disease (Bhat et al., 2012; Palmer et al., 2015; Jeon et al., 2017; Álvarez et al., 2017; Shimizu and Minamino, 2019) whose prognosis is influenced by emerging vascular damage. A slow pressor infusion of angiotensin II has been widely used as a model of chronic oxidative stress that will cause vascular damage and cellular senescence (Kunieda et al., 2006; Coffman, 2011; Wang et al., 2018). Here we employ conditional depletion of p16-dependent senescent cells in the INK-ATTAC model as a mechanistic approach to evaluate the contribution of senescence to the phenotype as well as cellular or molecular alterations.

Amongst the organs surveyed i.e., brain, lung and spleen, the kidneys were unique in showing p16^Ink4a^ activation after angiotensin II that was prevented by elimination of p16^Ink4a^ dependent, senescent cells using AP20187 (see Figure 1C). The ATTAC cassette contains a GFP coding sequence that is inserted downstream of an IRES allowing for staining and identification of cells that express GFP via the cassette (see Figure 1A). Interestingly, the most prominent staining for GFP appeared in glomerular and peritubular capillary networks (see Figure 3C). Staining for the endothelial cell specific von Willebrand factor (vWF) protein and mRNA after angiotensin II indicates a significant effect of angiotensin II on endothelia that is prevented by AP20187 (see Figures 3A,B). Staining of serial sections for vWF and GFP showed their cellular co-localization suggesting that the senescent cells in the kidney were of endothelial origin (Supplementary Figure S2B). Notably, other endothelial cell markers, CD31 (PECAM1) and CD34 mRNA reflecting the abundance of endothelia did not show a change (Carvalho et al., 1982; Fleming and Jones, 1989; Kinjo et al., 1989; Müller et al., 2002; Pusztaszeri et al., 2006; Westein et al., 2013; Dushpanova et al., 2016). Notably, vWF expression can be increased due to atherosclerosis (Westein et al., 2013) and hypertension (Dushpanova et al., 2016) and was recently reported to contribute to endotheliopathy from COVID-19 infection (Gu et al., 2020). Interestingly, expression of endothelial cell markers vary throughout tissues and organs, as endothelia in lungs solely express CD34 and CD31 but no vWF, in kidneys CD34, CD31 and focally vWF, in spleens and livers CD31 (Pusztaszeri et al., 2006). Here, in adjacent serial sections CD31 staining did not overlap with GFP or vWF in line with the expected heterogeneity of endothelia (Supplementary Figure S2B). Overall, the GFP and vWF co-staining suggests that senescent peritubular endothelial cells are expressing and secreting vWF and that based on staining and RNA expression, vWF expression decreases when those senescent cells are eliminated by AP20187 treatment.

The p21^Cip1^ and p15^Ink4b^ genes that are established indicators of senescence (Bernadesde Jesus and Blasco, 2012; Althubiti et al., 2014; Maciel-Baron et al., 2018), were significantly upregulated after angiotensin II, but prevented from upregulation by addition of AP20187. Interestingly, endogenous p16^Ink4a^ expression in the kidneys was below detection of the RNA-seq analysis and we thus can not comment on its regulation (data not shown). On the other hand, p15^Ink4b^ and p16^Ink4a^ expression effect an overlapping set of genes and p15^Ink4b^ was upregulated 4-fold by angiotensin II and returned to control by inclusion of AP20187 (see Figure 2B). Also, the findings from the Gene Set Enrichment Analysis (GSEA) that senescence pathways were enriched in the angiotensin II compared to the angiotensin II plus AP20187 group provides robust evidence of induction of senescence beyond individual signature genes that may be regulated in a tissue specific manner.

Interestingly, senescence associated secretory phenotype genes such as FGF2, MMP-3, IGFBP-2, and tPA were upregulated by angiotensin II and prevented by addition of AP20187 whereas p53 and other known SASP components, e.g., MMP-13, were not impacted. The selection of only certain genes of the SASP pool being upregulated aligns with recent findings where depending on the cell type and the senescence inducer, different members of the SASP may be induced (Wiley et al., 2017). This overall suggests a specific and selective response in the kidney environment (Coppe et al., 2010).

FGF2 is implicated in mitogenicity, angiogenesis, and differentiation (Shiba et al., 2003; Murakami and Simons, 2008; Nanda et al., 2014; Tassi et al., 2018) and interacts with angiotensin II to promote renal homeostasis (Peifley and Winkles, 1998; Fulgham et al., 1999). FGF2 may contribute to the appearance of growth factor signaling by GSEA where the PI3K-Akt-mTOR and angiogenesis pathways are significantly enriched in the angiotensin II and not in the angiotensin II plus AP20187 groups. Indeed FGF2 can induce vWF to promote angiogenesis (Peifley and Winkles, 1998; Fulgham et al., 1999; Kawecki et al., 2017). ANGPT2 also plays a role in angiogenesis towards promoting vascular destabilization (Kim et al., 2016; Kiss and Saharinen, 2017). Furthermore, as stated earlier, ANGPT2 and vWF are stored inside Weibel-Palade Bodies in endothelial cells and are released when the cells are activated or become dysfunctional (Kim et al., 2016; Gragnano et al., 2017; Kawecki et al., 2017; Kiss and Saharinen, 2017; Wiley et al., 2019).

Very relevant to our analysis, the RB1 pathway which is directly associated with p16^ink4a^ signaling showed significant (*p* < 0.001) 5.5-fold positive activation in the IPA gene expression analysis (Figure 1E; Supplementary Table S1). Downstream target genes of the RB1 pathway include ANGPT2 as discussed above. Also, mTOR signaling has been shown to associate with RB1 in that its activation allows for senescence progression and SASP secretion, while mTOR inhibition can lead to a change from senescence to quiescence and SASP inhibition (Korotchina et al., 2010). Interestingly, gene expression controlled by sirolimus, the well-known mTOR inhibitor, showed a highly significant (p = 4x10^−18^) 6.9-fold negative activation score in the IPA analysis (Figure 1E; Supplementary Table S1) in line with activation of the mTOR pathway during senescence induction.

One of the functions of the SASP is to recruit immune cells. As mentioned above, angiotensin II’s systemic impacts also include priming and activation of the immune system (Bengini et al., 2010; Guzik et al., 2007b; Nataraj et al., 1999b). Within the kidneys, specifically the renal glomerular and peritubular capillary network, we observed an increase in CD45R+ and CD3^+^ T cells that was reverted by addition of AP20187 (see Figures 4B,C) which is consistent with the recruitment of immune cells to senescent tissue compartments (Vicente et al., 2016). In addition, there was dramatic increase in mRNA expression of versican by angiotensin II that was reverted with the addition of AP20187. Versican is an extracellular matrix proteoglycan that is produced by stromal cells, smooth muscle cells, and fibroblasts during inflammation and promotes adhesion of white blood cells resulting in enhancement of the inflammatory response (Wight et al., 2014). A recent study has also shown that versican can be produced by endothelial cells during extreme stress (i. e., hyperglycemia) (Li et al., 2019). Notably, elimination of the senescent cells by AP20187 removes the inflammatory phenotype providing a mechanistic explanation for the phenotype.

Finally, in the current study the blood pressure was increased in the angiotensin II group although only slightly as expected from the low dose angiotensin II regimen and the addition of AP20187 to the angiotensin II treatment prevented an increase (Supplementary Figure S1A, Supplementary Figure S1B). It is noteworthy that chronic AP20187 treatment alone did not alter blood pressure relative to a control treatment (data not shown).

**FIGURE 5:**
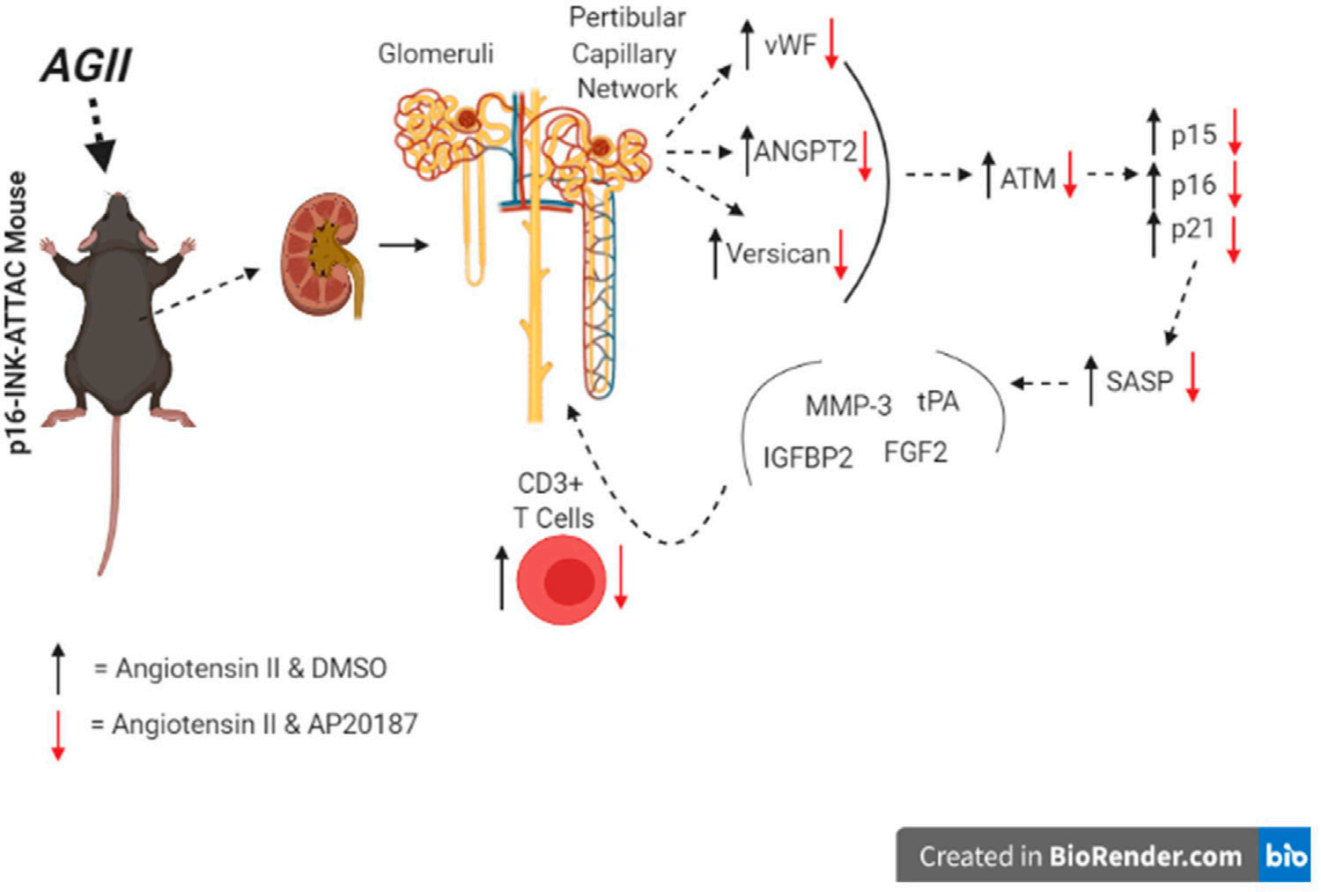
Overview of pathways impacted in kidneys (graphical abstract).

In conclusion, the low dose, prolonged angiotensin II exposure is associated with the induction of senescence in kidneys and the promotion of an inflammatory microenvironment through both secreted factors and immune cells (Figure 5). Endothelial cells appear to be a major cell type impacted. The elimination of senescent cells in the INK-ATTAC transgenic model prevents these effects of angiotensin II and reveals a novel pathophysiologic mechanism amenable to targeting by senolytic drugs in development.

## Limitations

A potential limitation in this study is that the utilization of AP20187 in the INK-ATTAC model only eliminates senescent cells induced by p16^Ink4a^. From the RNA-seq analysis, there was also increased expression of p21 and p15 and we were surprised that their increased expression was also reversed after AP20187. The most likely explanation is that several parallel pathways are turned on in cells undergoing senescence-inducing damage and that those cells are eliminated due to the activation of p16. Though less likely, it is conceivable that the paracrine activity of senescent cells from p16^Ink4a^ expressing cells may cause induction of p21 and p15 in surrounding cells. With the elimination of the p16^Ink4a^ senescent cells, p21 and p15 expression would be reverted.

## Data Availability

The datasets presented in this study can be found in online repositories. The names of the repository/repositories and accession number(s) can be found below: GEO repository number GSE179195.

## Supplementary Material

Supplementary Figure S1 Changes in blood pressure relative to baseline. Blood pressure at baseline and after angiotensin II **(A)** and angiotensin II plus AP20187 **(B)**. Solid lines indicate increase, thin lines decrease, dotted lines no change.

Supplementary Figure S2 Senescence and Endothelial Cell Staining. **(A)** Senescence associated Beta-Galactosidase Staining after angiontensin II and angiotensin II plus AP20187. Glomeruli indicated by dotted circle **(B)** Staining of adjacent serial sections (5 µm thick) of kidneys from angiotensin II treated mice for GFP, vWF, and CD31. The green arrows in the GFP and vWF stained sections indicate the same region. The green arrow in the CD31 stained section indicates a positively stained endothelial cell. Scale Bars are 0.1 mm.

Supplementary Table S1 RNA-seq gene expression analysis of kidneys. Ingenuity Pathway Analysis was used to identify Upstream Regulators. P-values and activation z-scores are shown. Negative z-scores indicate inhibitory Upstream Regulators. Fold changes of expression values for individual genes are included when detected. The RNAseq data are deposited at GEO under GSE179195.

Supplementary Table S2 RNA-seq gene expression analysis of kidneys using Gene Set Enrichment Analysis for Hallmark and Gene Ontology Pathways. Size, Normalized Enrichment Score (NES), Enrichment Score (ES), nominal p-values, false discovery corrected p-values, and q-values are shown. The RNAseq data are deposited at GEO under GSE179195.
