# Supplementary material for: Low Dose Chronic Angiotensin II Induces Selective Senescence of Kidney Endothelial Cells
Source: Front Cell Dev Biol. 2021 Dec 8;9:782841. doi: 10.3389/fcell.2021.782841 (PMC8696590; doi:10.3389/fcell.2021.782841)
Supplement: Supplementary file 1 [file Table1.pdf]

# Table S1

| Master Regulator | Expr Log Ratio | Activation z-score | p-value of overlap |
|---|---|---|---|
| PFDN5 | 0.785 | -8.124 | 3.95E-23 |
| FBXL14 | -0.104 | -7.347 | 1.19E-24 |
| estrogen | NA | -7.316 | 3.05E-25 |
| CD 437 | NA | -7.208 | 1.99E-16 |
| SGI 1776 | NA | -7.1 | 2.96E-24 |
| debio 1347 | NA | -7.038 | 7E-25 |
| derazantinib FGFR KI | NA | -7.038 | 7E-25 |
| pemigatinib FGFR KI | NA | -7.038 | 7E-25 |
| GABPB1 | -0.139 | -7.02 | 5.01E-29 |
| ST1926 | NA | -7.02 | 8.58E-16 |
| procaine | NA | -6.908 | 3.33E-23 |
| sirolimus | NA | -6.902 | 4.28E-18 |
| 5-fluorouracil | NA | -6.696 | 5.26E-15 |
| ASP5878 | NA | -6.665 | 2.64E-25 |
| Erdafitinib | NA | -6.665 | 2.64E-25 |
| 3D185 | NA | -6.602 | 6.3E-26 |
| ARG1 | 1.866 | -6.528 | 1.43E-27 |
| PRN1371 | NA | -6.465 | 3.34E-25 |
| GW 273629 | NA | -6.229 | 3.32E-32 |
| nicotine | NA | -6.225 | 5.71E-25 |
| Tripterygium wilfordii extract | NA | -6.224 | 5.21E-30 |
| FBXO32 | -1.097 | -6.027 | 2.55E-25 |
| S-methylthiocitrulline | NA | -5.95 | 7.37E-31 |
| KDM5A | -0.665 | -5.729 | 1.76E-11 |
| RBL1 | -0.343 | -5.565 | 1.08E-32 |
| RRAD | 0.086 | -5.498 | 0.0000273 |
| vinorelbine | NA | -5.455 | 2.02E-14 |
| alisertib | NA | -5.436 | 1.17E-26 |
| DEGS1 | 0.148 | -5.334 | 0.0000305 |
| BACH1 | -0.452 | -5.178 | 2.03E-08 |
| enalapril ACE inhib | NA | -5.138 | 1.09E-23 |
| cinaciguat | NA | -5.124 | 4.18E-11 |
| TLX1 | NA | -5.112 | 1.11E-08 |
| HUWE1 | -0.812 | -5.046 | 2.5E-24 |
| FBXL14 | -0.104 | -4.838 | 1.6E-30 |
| CRMP1 | -0.452 | -4.73 | 1.14E-24 |
| FBXW7 | -0.145 | -4.683 | 8.24E-27 |
| TRIM28 | 0.022 | -4.587 | 3.18E-26 |
| Danusertib | NA | -4.537 | 1.33E-28 |
| atorvastatin | NA | -4.493 | 2.7E-23 |
| KDM5B | -0.612 | -4.472 | 0.0000105 |
| ABT-089 | NA | -4.445 | 5.58E-24 |
| PFDN5 | 0.785 | -4.431 | 4.86E-28 |
| Candesartan | NA | -4.424 | 2.2E-23 |
| AMG 900 | NA | -4.406 | 1.7E-27 |
| TTP607 | NA | -4.406 | 1.7E-27 |
| ACP-319 | NA | -4.379 | 2.53E-25 |
| SNS 314 | NA | -4.363 | 2.73E-27 |
| SIRT6 | 0.238 | -4.259 | 1E-34 |
| ELOVL5 | -0.205 | -4.206 | 4.55E-09 |
| KDM2B | -0.508 | -4.196 | 0.00000183 |

| | | | |
|---|---|---|---|
| resveratrol | NA | -4.146 | 2.9E-23 |
| BRCA1 | -1.262 | -4.103 | 4.33E-25 |
| PML-RARA | NA | -4.098 | 0.00000248 |
| RND3 | 0.124 | -4.071 | 1.01E-32 |
| MSRA | 0.445 | -4.061 | 0.00000218 |
| rabeprazole | NA | -3.994 | 4.73E-26 |
| BRL 26830 | NA | -3.969 | 1.75E-13 |
| YY1 | -0.266 | -3.961 | 1.38E-24 |
| flumatinib | NA | -3.902 | 4.39E-26 |
| radotinib | NA | -3.761 | 1.71E-24 |
| KEAP1 | -0.057 | -3.674 | 0.00000121 |
| LEP | -1.215 | -3.669 | 9.62E-20 |
| MXD1 | -0.057 | -3.658 | 1.59E-28 |
| CPT1B | NA | -3.58 | 5.1E-24 |
| warfarin | NA | -3.569 | 6.18E-24 |
| SIRT7 | 0.509 | -3.516 | 4.08E-26 |
| Sb202190 | NA | -3.503 | 1.19E-18 |
| GNA12 | -0.158 | -3.5 | 0.00314 |
| S-equol | NA | -3.474 | 3.77E-24 |
| BACH1 | -0.452 | -3.441 | 0.00000624 |
| WNT1 | NA | -3.432 | 3.64E-16 |
| HOXA10 | 0.218 | -3.402 | 1.46E-29 |
| STAT5A | -0.443 | -3.374 | 9.52E-24 |
| BIX 02189 | NA | -3.366 | 2.1E-31 |
| SRP54 | -0.834 | -3.342 | 4.45E-19 |
| halothane | NA | -3.317 | 6.47E-15 |
| AKR1B1 | -1.963 | -3.242 | 4.39E-21 |
| PIM/FLT3 kinase inhibitor SEL24 | NA | -3.229 | 3.5E-25 |
| deoxycorticosterone acetate salt sens hypertension | NA | -3.208 | 1.97E-17 |
| ECD | -0.028 | -3.186 | 1.46E-19 |
| CL 316243 | NA | -3.162 | 0.000628 |
| phenylephrine | NA | -3.154 | 1.86E-17 |
| CCL7 | NA | -3.108 | 0.00000364 |
| PPIF | 0.646 | -3.108 | 2.15E-23 |
| GSK690693 | NA | -3.101 | 0.00000418 |
| HOXB1 | NA | -3.077 | 1.91E-27 |
| LMO2 | 0.305 | -3.069 | 1.64E-23 |
| sepantronium | NA | -3.064 | 6.16E-27 |
| RGS2 | -0.16 | -2.985 | 0.00133 |
| CCL25 | 0.074 | -2.959 | 4.21E-08 |
| papaverine | NA | -2.937 | 0.00000035 |
| PCGF2 | 0.379 | -2.889 | 0.00000705 |
| PTGDS | -0.245 | -2.887 | 0.00000131 |
| ruxolitinib | NA | -2.887 | 0.00000868 |
| SKI-G-801 | NA | -2.881 | 2.42E-26 |
| RAP1B | 0.104 | -2.874 | 2.21E-08 |
| BMS-345541 | NA | -2.86 | 3.64E-29 |
| CDC42 | NA | -2.86 | 3.6E-18 |
| YY1 | -0.266 | -2.844 | 1.85E-26 |
| TRAP1 | 0.082 | -2.837 | 0.00136 |
| valproic acid | NA | -2.75 | 0.0000379 |
| SAR1B | 0.076 | -2.73 | 1.73E-19 |

| | | | |
|---|---|---|---|
| MICAL1 | 0.443 | -2.707 | 3.8E-23 |
| PD184352 | NA | -2.704 | 7.31E-20 |
| chelerythrine | NA | -2.699 | 1.25E-14 |
| pimagedine | NA | -2.692 | 6.34E-19 |
| l-asparaginase | NA | -2.673 | 0.000000259 |
| ghrelin | NA | -2.668 | 0.0000714 |
| interferon beta-1a | NA | -2.654 | 4.79E-18 |
| TP73 | NA | -2.648 | 3.63E-20 |
| PISD | 0.374 | -2.646 | 0.00104 |
| eplerenone | NA | -2.635 | 0.0000158 |
| IDH1 | 0.216 | -2.6 | 7.15E-08 |
| RNF139 | 0.207 | -2.596 | 1.91E-30 |
| oseltamivir | NA | -2.573 | 7.85E-12 |
| NDN | -0.398 | -2.566 | 0.0000553 |
| ERG | -0.225 | -2.556 | 0.000172 |
| St. John's wort | NA | -2.54 | 1.52E-20 |
| ACTN4 | -0.129 | -2.525 | 0.0000115 |
| FUT6 | NA | -2.525 | 0.000000811 |
| apomorphine | NA | -2.514 | 0.000000193 |
| SIRT3 | 0.315 | -2.5 | 0.000506 |
| PAFAH1B1 | -0.279 | -2.498 | 1.57E-22 |
| TRIM28 | 0.022 | -2.498 | 5.08E-29 |
| dalteparin | NA | -2.496 | 0.000409 |
| E2F3 | 0.154 | -2.478 | 5.63E-15 |
| CREG1 | 0.338 | -2.462 | 0.0000915 |
| PPT1 | 0.122 | -2.462 | 0.0000915 |
| sobetirome | NA | -2.449 | 0.0000758 |
| ST8SIA1 | -0.308 | -2.449 | 0.0249 |
| GGT1 | 0.14 | -2.447 | 6.74E-16 |
| SOX4 | -0.56 | -2.44 | 0.000000267 |
| alvespimycin | NA | -2.429 | 0.00000196 |
| S-equol | NA | -2.428 | 3.13E-16 |
| TP53BP1 | -0.363 | -2.428 | 6.42E-27 |
| BI 847325 | NA | -2.4 | 6.04E-08 |
| HOXA1 | 0.853 | -2.394 | 0.000000661 |
| methadone | NA | -2.384 | 1.08E-17 |
| HPRT1 | 0.071 | -2.38 | 2.34E-14 |
| GW501516 | NA | -2.357 | 0.00000418 |
| prazosin | NA | -2.357 | 1.56E-15 |
| policosanol | NA | -2.355 | 7.88E-24 |
| HOXA10 | 0.218 | -2.333 | 0.000175 |
| ZFP36 | 1.761 | -2.333 | 0.0000365 |
| HOXA4 | -0.264 | -2.324 | 0.000000268 |
| diazepam | NA | -2.321 | 0.00000655 |
| PIN4 | 0.926 | -2.321 | 0.0000842 |
| FGF9 | 0.519 | -2.292 | 1.23E-09 |
| STAT5B | -0.357 | -2.292 | 0.000002 |
| ASAH2 | -0.518 | -2.25 | 0.000077 |
| NUS1 | -0.136 | -2.25 | 0.000128 |
| PDE2A | NA | -2.242 | 6.51E-19 |
| pitavastatin | NA | -2.242 | 2.63E-08 |
| firsocostat | NA | -2.236 | 0.0000351 |

| | | | |
|---|---|---|---|
| HOXA4 | -0.264 | -2.236 | 0.00000882 |
| pertuzumab | NA | -2.236 | 4.09E-12 |
| propylthiouracil | NA | -2.236 | 0.0000258 |
| SAT1 | 0.611 | -2.236 | 0.0107 |
| vinorelbine | NA | -2.236 | 0.0152 |
| GNL2 | 0.224 | -2.233 | 1.04E-12 |
| LIPE | -1.448 | -2.228 | 2.89E-08 |
| bezafibrate | NA | -2.227 | 9.02E-08 |
| HOXA5 | -0.21 | -2.223 | 2.24E-23 |
| pyrilamine | NA | -2.219 | 1.5E-14 |
| GnRH analog | NA | -2.213 | 0.000163 |
| almotriptan | NA | -2.209 | 3.12E-09 |
| pregnenolone carbonitrile | NA | -2.2 | 1.49E-19 |
| PND-1186 | NA | -2.183 | 0.000000774 |
| PHLPP2 | -0.634 | -2.18 | 1.62E-11 |
| IGF1 | -0.316 | -2.176 | 1.92E-19 |
| PELI1 | -0.036 | -2.155 | 1.46E-22 |
| tetraethylammonium | NA | -2.138 | 0.0000695 |
| sorafenib analog BB2 | NA | -2.121 | 1.97E-13 |
| BCAR1 | 0.081 | -2.111 | 2.1E-10 |
| FAAH | 0.132 | -2.111 | 0.0000562 |
| silicon dioxide | NA | -2.108 | 3.9E-20 |
| beta-escin | NA | -2.092 | 2.26E-28 |
| dihematoporphyrin ether | NA | -2.092 | 3.31E-28 |
| N-Ac-Leu-Leu-norleucinal | NA | -2.078 | 1.09E-26 |
| FASN | -4.417 | -2.065 | 0.0000415 |
| valpromide | NA | -2.05 | 4.16E-17 |
| DNM2 | -0.037 | -2.034 | 3.91E-15 |
| inolitazone | NA | -2.03 | 1.07E-09 |
| BDTX-189 | NA | -2.023 | 7.76E-13 |
| sapitinib | NA | -2.023 | 7.76E-13 |
| CGAS | -0.487 | -2.02 | 3.87E-16 |
| rigosertib PLK1 / PI3K/Akt inhib | NA | -2.02 | 0.000000427 |
| STAT5A | -0.443 | -2.019 | 9.91E-18 |
| SIRT1 inhibitor III | NA | -2.018 | 4.64E-09 |
| fludrocortisone | NA | -2.01 | 1.11E-22 |
| continuous erythropoietin receptor activator | NA | -2 | 0.000108 |
| FH | 0.288 | -2 | 0.002 |
| MSH2 | -0.01 | -2 | 0.0000273 |
| pentobarbital | NA | -2 | 0.000199 |
| ST8SIA4 | -0.555 | -2 | 0.000117 |
| TXNRD1 | -0.081 | -2 | 0.0000061 |
| ACACB | -1.535 | 2 | 0.000199 |
| DNMT3A | 0.038 | 2 | 0.000000233 |
| namitecan | NA | 2 | 0.0193 |
| pCPT-cAMP | NA | 2 | 0.000448 |
| PFDN5 | 0.785 | 2 | 0.002 |
| PT-2385 | NA | 2 | 0.00426 |
| zileuton | NA | 2.012 | 0.0000242 |
| AZD6482 | NA | 2.014 | 8.8E-09 |
| EGF | 0.575 | 2.03 | 1.63E-13 |
| EAF2 | 0.403 | 2.058 | 0.000000139 |

| | | | |
|---|---|---|---|
| SP600125 | NA | 2.076 | 5.2E-17 |
| FKBP4 | 0.166 | 2.093 | 5.14E-18 |
| iohexol | NA | 2.109 | 0.0000915 |
| antilymphocyte serum | NA | 2.111 | 0.00000272 |
| SESN2 | -0.406 | 2.115 | 1.32E-08 |
| ID3 | 0.745 | 2.12 | 1.89E-08 |
| PRKN | 0.18 | 2.121 | 0.0272 |
| STUB1 | 0.013 | 2.121 | 0.0000394 |
| SPDEF | NA | 2.138 | 0.00434 |
| chlorogenic acid | NA | 2.155 | 5.2E-21 |
| ANG | 0.478 | 2.165 | 3.15E-24 |
| FTO | -0.155 | 2.173 | 1.36E-08 |
| PRPF6 | -0.038 | 2.179 | 1.09E-18 |
| TRRAP | -0.699 | 2.186 | 7.32E-29 |
| BHLHA15 | NA | 2.187 | 9.68E-12 |
| atorvastatin | NA | 2.2 | 2.89E-12 |
| clozapine | NA | 2.226 | 0.00000125 |
| 4-hydroxytamoxifen | NA | 2.236 | 2.2E-09 |
| ANGPTL3 - coincides with ANGPT2 exp | 0.331 | 2.236 | 0.000758 |
| GRP | NA | 2.236 | 0.000335 |
| LIF | NA | 2.236 | 4.8E-09 |
| PAX8 | 0.131 | 2.236 | 0.0000162 |
| bisindolylmaleimide iv | NA | 2.243 | 2.05E-18 |
| Doxorubicin | NA | 2.249 | 1.18E-26 |
| ENPP2 | -0.173 | 2.25 | 0.000117 |
| GSK2636771 | NA | 2.25 | 0.0000913 |
| TGX-221 | NA | 2.25 | 0.000128 |
| TNKS2 | -0.294 | 2.258 | 2.6E-12 |
| TNKS | -0.679 | 2.258 | 2.09E-12 |
| AT13148 | NA | 2.287 | 0.0000604 |
| tegaserod | NA | 2.29 | 5.63E-22 |
| ABTL0812 | NA | 2.309 | 0.00706 |
| BI 860585 | NA | 2.309 | 0.00706 |
| DS-3078a | NA | 2.309 | 0.00706 |
| LXI-15029 | NA | 2.309 | 0.00706 |
| finasteride | NA | 2.312 | 0.0000104 |
| MYCN | -0.204 | 2.313 | 4.23E-21 |
| CREM | 0.082 | 2.324 | 3.05E-09 |
| dec-RVKR-CMK | NA | 2.325 | 0.0000222 |
| HIC1 | -0.305 | 2.325 | 0.0000144 |
| prexasertib | NA | 2.333 | 0.0000206 |
| HNF4A | -0.007 | 2.335 | 1.15E-08 |
| barbiturate | NA | 2.357 | 5.58E-09 |
| pergolide | NA | 2.408 | 9.51E-16 |
| cyclosporin A | NA | 2.425 | 0.0000432 |
| wortmannin - inhibits DNA repair | NA | 2.429 | 4.15E-21 |
| temsirolimus | NA | 2.44 | 0.000000244 |
| PCYT2 | 0.628 | 2.449 | 0.00601 |
| MSC2363318A | NA | 2.462 | 0.0000842 |
| azathioprine | NA | 2.496 | 1.06E-14 |
| NRF1 | -0.02 | 2.5 | 0.00135 |
| FGF19 | NA | 2.53 | 0.000862 |

| | | | |
|---|---|---|---|
| TAF4 | 0.025 | 2.546 | 4.92E-17 |
| FOXO3 - senescence inducer | -0.645 | 2.565 | 8.36E-15 |
| ALOX5 | -0.197 | 2.61 | 4.36E-26 |
| MBOAT7 | 0.002 | 2.646 | 0.0000502 |
| fluoxetine | NA | 2.65 | 2.79E-17 |
| dutasteride | NA | 2.654 | 0.0000195 |
| tyrphostin AG 1478 | NA | 2.657 | 3.62E-17 |
| bromodeoxyuridine | NA | 2.66 | 1.03E-24 |
| stallimycin - DNA damage inducer | NA | 2.664 | 2.15E-24 |
| NRF1 - antioxidant | -0.02 | 2.668 | 0.000812 |
| PDGF receptor tyrosine kinase inhibitor IV | NA | 2.668 | 0.0000714 |
| gemopatrilat | NA | 2.677 | 2.98E-11 |
| omapatrilat | NA | 2.677 | 2.98E-11 |
| thiorphan | NA | 2.677 | 4.5E-11 |
| AZD8055 | NA | 2.708 | 2.52E-09 |
| Esrra | 0.245 | 2.711 | 0.000542 |
| adaphostin | NA | 2.736 | 4.6E-16 |
| PITX2 | -0.698 | 2.746 | 0.000765 |
| RUNX2 | -0.119 | 2.817 | 8.94E-12 |
| captopril - ACE inhibitor | NA | 2.828 | 0.00225 |
| FGF19 | NA | 2.828 | 0.000000155 |
| MARCHF8 | -0.048 | 2.828 | 1.24E-20 |
| FOXO1 | -0.471 | 2.853 | 0.0000773 |
| mirdametinib MEK inh | NA | 2.862 | 2.58E-19 |
| EHD4 | -0.387 | 2.87 | 8.14E-31 |
| bardoxolone methyl - inhibits NfKB and thus SASP | NA | 2.878 | 5.19E-14 |
| dalfampridine | NA | 2.887 | 6.43E-09 |
| LMO4 | 0.483 | 2.959 | 1.88E-23 |
| CREB1 | -0.154 | 2.967 | 2.03E-14 |
| PRL | NA | 2.994 | 0.0000332 |
| HES5 | NA | 3.011 | 1.79E-22 |
| teriflunomide | NA | 3.029 | 0.000000109 |
| BT2 | NA | 3.051 | 0.00000427 |
| AZD-7451 | NA | 3.077 | 4.52E-13 |
| CREBBP | -0.429 | 3.118 | 2.49E-27 |
| indinavir | NA | 3.154 | 4.23E-10 |
| misoprostol | NA | 3.197 | 1.62E-24 |
| SP4 | -0.54 | 3.266 | 3.35E-23 |
| bexarotene | NA | 3.286 | 1.55E-08 |
| curcumin | NA | 3.289 | 5.82E-19 |
| everolimus | NA | 3.311 | 3.83E-15 |
| fenoterol (promote renin release) | NA | 3.311 | 1.62E-14 |
| AREG | NA | 3.317 | 0.00000343 |
| mycophenolic acid | NA | 3.341 | 6.99E-09 |
| WWTR1 | -0.209 | 3.384 | 6.33E-17 |
| simvastatin | NA | 3.421 | 8.61E-18 |
| PF4 | 0.751 | 3.464 | 4.09E-10 |
| OSI-461 | NA | 3.485 | 2.36E-24 |
| captopril | NA | 3.544 | 5.49E-08 |
| eflornithine | NA | 3.727 | 2.52E-08 |
| ZIC5 | NA | 3.735 | 1.1E-24 |
| RAD21 | -0.294 | 3.788 | 1.68E-30 |

| | | | |
|---|---|---|---|
| pantoprazole | NA | 3.795 | 0.00000115 |
| TRERF1 | -0.167 | 3.796 | 0.00000071 |
| BCAT1 | -2.297 | 3.807 | 1.44E-08 |
| CINK4 | NA | 3.878 | 0.000000198 |
| cloprostenol | NA | 3.881 | 1E-24 |
| SIRT3 | 0.315 | 3.903 | 2.04E-20 |
| lycopene | NA | 3.973 | 1.65E-15 |
| posaconazole | NA | 4.01 | 9.96E-08 |
| 4-hydroxytamoxifen | NA | 4.028 | 3.52E-29 |
| KLF3 | -0.318 | 4.036 | 0.000248 |
| cholestyramine | NA | 4.162 | 3.87E-24 |
| IDEC-132 | NA | 4.185 | 9.27E-16 |
| NFE2L2 - antioxidant | 0.2 | 4.196 | 0.00000372 |
| ZMIZ1 | -0.328 | 4.233 | 1.38E-28 |
| BCR-ABL1 | NA | 4.302 | 6.43E-23 |
| CDX1 | NA | 4.308 | 7.61E-08 |
| ALDH3A1 - metabolizes ROS byproducts | -3.105 | 4.388 | 0.0000206 |
| cisatracurium | NA | 4.456 | 1.88E-23 |
| pipecuronium | NA | 4.456 | 1.88E-23 |
| PARP16 | 0.209 | 4.491 | 4.3E-21 |
| CGP 74514A | NA | 4.571 | 6.47E-11 |
| MYC | 0.114 | 4.579 | 1.05E-24 |
| blinatumomab | NA | 4.65 | 4.69E-28 |
| MYCL | -0.773 | 4.707 | 0.00000163 |
| rocuronium | NA | 4.807 | 4.72E-25 |
| BTG2 | -0.114 | 5.014 | 3.05E-10 |
| SCGB3A1 | NA | 5.199 | 2.89E-08 |
| acyclic retinoid | NA | 5.246 | 2.15E-10 |
| EPAS1 | -0.318 | 5.288 | 2.4E-27 |
| fulvestrant | NA | 5.331 | 4.36E-17 |
| Slfn1 | NA | 5.361 | 1.26E-09 |
| DDX5 | 0.322 | 5.396 | 9.96E-18 |
| SU 9516 | NA | 5.409 | 9.05E-09 |
| RB1 - senescence | -0.465 | 5.506 | 0.0000525 |
| enflurane | NA | 5.541 | 1.52E-22 |
| epothilone B | NA | 5.578 | 0.0000276 |
| PAPOLA | -0.359 | 5.588 | 0.0000404 |
| doxazosin - alpha blocker | NA | 5.735 | 0.0000155 |
| PAX5 | 1.508 | 5.979 | 1.88E-13 |
| IKZF1 | -0.092 | 6.136 | 7.1E-13 |
| ERG | -0.225 | 6.174 | 1.49E-24 |
| sapropterin | NA | 6.229 | 3.32E-32 |
| NOS2 | -0.057 | 6.695 | 3.74E-27 |
| DEK-NUP214 | NA | 7.068 | 2.84E-23 |
| enzastaurin | NA | 7.178 | 6.96E-23 |
| MLXIPL | 0.185 | 7.333 | 4.13E-57 |
| MYCN | -0.204 | 7.509 | 2.66E-19 |
| MYC | 0.114 | 7.535 | 1.86E-22 |
| TRRAP | -0.699 | 7.636 | 8.76E-23 |
