# Supplementary material for: Low Dose Chronic Angiotensin II Induces Selective Senescence of Kidney Endothelial Cells
Source: Front Cell Dev Biol. 2021 Dec 8;9:782841. doi: 10.3389/fcell.2021.782841 (PMC8696590; doi:10.3389/fcell.2021.782841)
Supplement: Supplementary file 2 [file Table2.pdf]

Table S2

NAME	SIZE	ES	NES	NOM p-val	FDR q-val	FWER p-val	RANK AT MAX	LEADING EDGE
HALLMARK_PI3K_AKT_MTOR_SIGNALING	97	0.418061	1.329381	0.26403326	0.15828758	0.172	2540	tags=21%, list=19%, signal=25%
HALLMARK_ANGIOGENESIS	28	0.740416	1.344968	0	0.12538557	0.131	489	tags=14%, list=4%, signal=15%
HALLMARK_APICAL_JUNCTION	175	0.659711	1.263429	0	0.21652801	0.463	1863	tags=23%, list=14%, signal=27%
HALLMARK_EPITHELIAL_MESENCHYMAL_TRANSITION	174	0.62185	1.256209	0	0.25107184	0.463	1661	tags=29%, list=13%, signal=33%
GO_INTERFERON_GAMMA_MEDIATED_SIGNALING_PATHWAY	62	0.698833	1.297667	0	0.18856429	1	1539	tags=26%, list=12%, signal=29%
GO_CELLULAR_SENESCENCE	54	0.487435	1.300857	0	0.18289772	0.952	1921	tags=17%, list=15%, signal=19%
GO_RENAL_SYSTEM_PROCESS_INVOLVED_IN_REGULATION_OF_SYSTEMIC_ARTERIAL_BLOOD_PRESSURE	18	0.425738	1.26993	0.26789367	0.2156871	1	1576	tags=11%, list=12%, signal=13%
