# Supplementary figures and images for: Low Dose Chronic Angiotensin II Induces Selective Senescence of Kidney Endothelial Cells
Source: Front Cell Dev Biol. 2021 Dec 8;9:782841. doi: 10.3389/fcell.2021.782841 (PMC8696590; doi:10.3389/fcell.2021.782841)

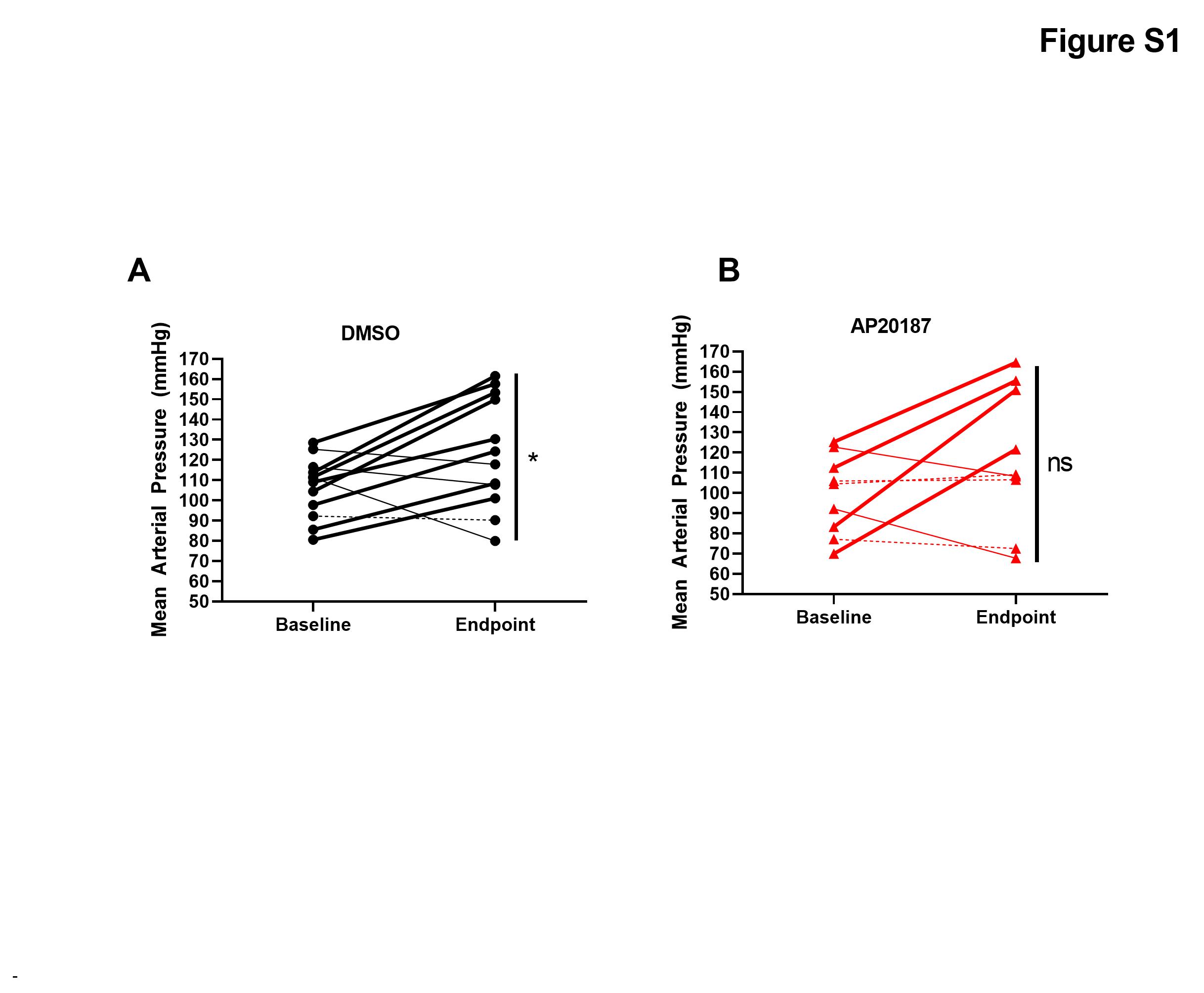

Supplement: Supplementary file 3 [file Image1.JPEG]

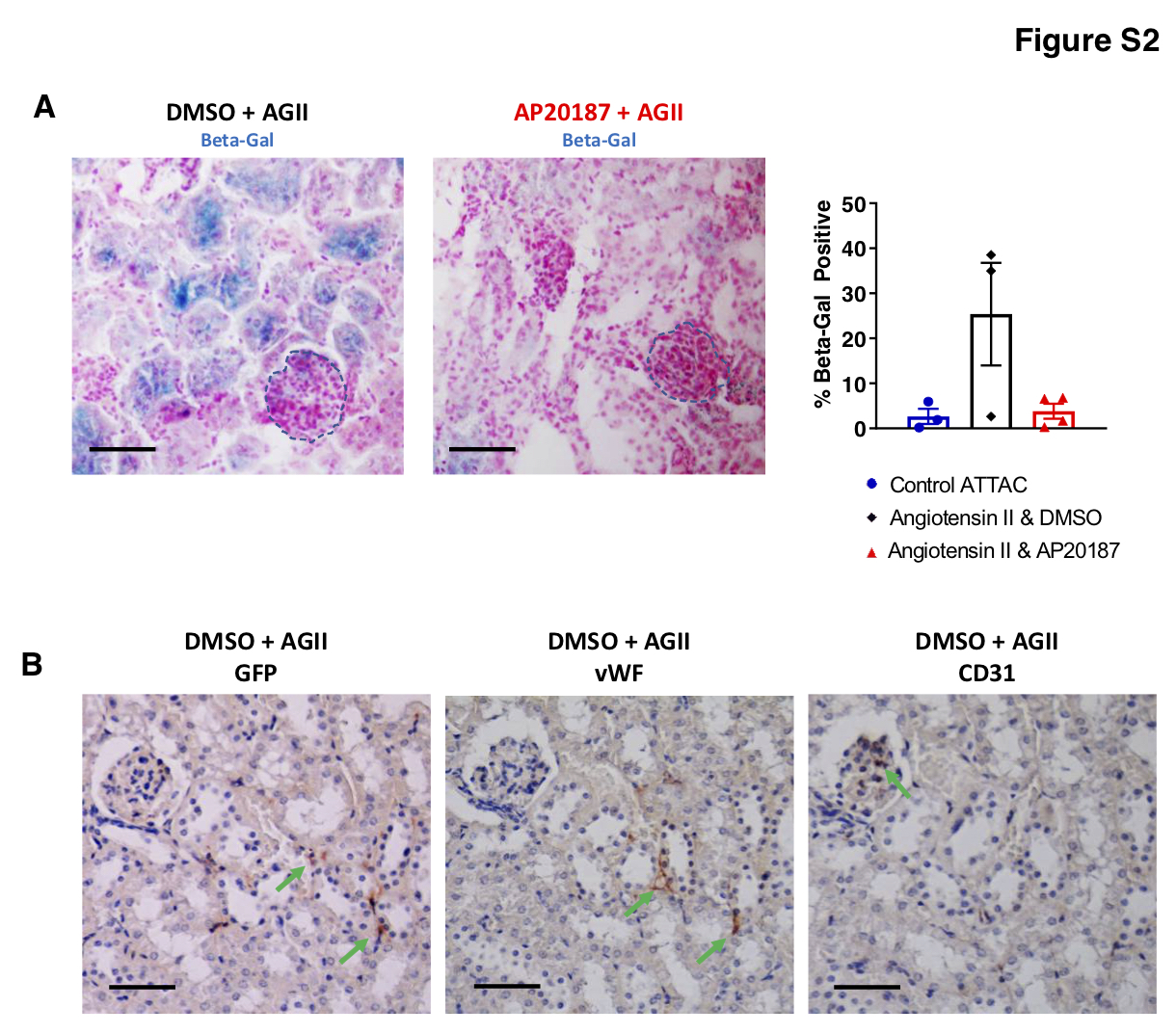

Supplement: Supplementary file 4 [file Image2.JPEG]
